# Pharmacological and resting state fMRI reveal Osteocalcin’s effects on mouse brain regions with high *Gpr37* and *Gpr158* expression

**DOI:** 10.1038/s41598-025-95000-2

**Published:** 2025-03-24

**Authors:** Natalia K. Freus, Isabel Wank, Maximilian Häfele, Liubov S. Kalinichenko, Christian P. Müller, Sandra Strobelt, Andreas Ludwig, Andreas Hess, Silke Kreitz

**Affiliations:** 1 Institute of Experimental and Clinical Pharmacology and Toxicology, Friedrich-Alexander-University Erlangen-Nuremberg, Fahrstraße 17, 91054 Erlangen, Germany; 2 Department of Psychiatry and Psychotherapy, University Hospital, Friedrich-Alexander-University Erlangen-Nuremberg, Schwabachanlage 6, 91054 Erlangen, Germany; 3 Central Institute for Mental Health, J5, 68159 Mannheim, Germany; 4 Institute of Neuroradiology, University Hospital Erlangen, Friedrich-Alexander-University Erlangen-Nuremberg, Schwabachanlage 6, 91054 Erlangen, Germany; 5 FAU NeW – Research Center for New Bioactive Compounds, Friedrich-Alexander- University Erlangen-Nuremberg, Erlangen, Germany

**Keywords:** Osteocalcin, Mouse fMRI, Functional connectivity, rCBV, Depression, Allen brain atlas, Neuroscience, Cognitive neuroscience, Diseases of the nervous system, Genetics of the nervous system, Neural circuits, Anxiety, Depression

## Abstract

Osteocalcin (OCN) is an endocrine hormone that signals in the periphery, regulating male fertility, energy expenditure and glucose homeostasis. It can also cross the blood-brain-barrier and act on the brain via receptors GPR37 and GPR158. In the brain, OCN influences neurotransmitter synthesis of serotonin, norepinephrine, and dopamine. OCN’s function is related to cognitive and memory performance and lack of OCN is associated with anxiety and depression-like behavior in mice. We used multiparametric magnetic resonance imaging (MRI) including pharmacological MRI and resting state functional MRI, along with gene expression data for *Gpr37* and *Gpr158* to investigate the physiological effects of intravenously administered OCN on the wild type mouse brain. We found four core brain regions (brainstem, limbic output, association cortex, and basal ganglia) that are highly relevant in all three analytical modalities (i.e. pharmacological, resting state MRI and gene expression) and play therefore a major role in mediating OCN’s effect in the brain. This study provides the first imaging data of the physiological impact of OCN on the mouse brain, suggesting its potential role in modulating brain function and its relevance as a candidate for further investigation in anxiety, depression, and cognitive impairments.

**Supplementary Information:**

The online version contains supplementary material available at 10.1038/s41598-025-95000-2.

## Introduction

Osteocalcin (OCN) is an endocrine hormone which is produced by osteoblasts and is primarily known for its function in bone formation by binding to hydroxyapatite in its carboxylated form^1^. The undercarboxylated form of OCN (in the following only referred to as ‘OCN’ unless stated otherwise) regulates various processes such as male fertility^2^, energy regulation in the skeletal muscle^3^, and insulin secretion in the pancreas^4,5^. These peripheral actions are mediated by the G-protein coupled receptor GPRC6A^4–6^. OCN’s endocrine functions further extend to the brain, as it can cross the blood–brain barrier (BBB)^7^. It is involved in the regulation of the synthesis of neurotransmitters such as gamma-aminobutyric acid (GABA), serotonin (5-HT), dopamine, and norepinephrine (NE)^7^. OCN knockout (OCN^-/-^) mice show elevated levels of GABA, whereas monoamine neurotransmitter contents in the brain are decreased. OCN^-/-^ mice exhibit anxiety and depression-like behaviors as well as reduced motor-exploratory activity^7^. As decreased levels of 5-HT and NE at key sites in the brain can lead to depression in humans (monoamine-deficiency hypothesis)^8^, depression-like behavior in OCN^-/-^ mice might occur due to decreased monoamine levels. Moreover, OCN also appears to regulate learning and memory, since OCN^-/-^ mice show significant spatial learning deficits in the Morris Water Maze test. These deficits were attributed to OCN’s role in hippocampal neurogenesis^7^.

The above findings raised the question if OCN could potentially be used as treatment for anxiety, depression-like behavior, and cognitive defects. One preclinical study using OCN^-/-^ mice showed that intracerebroventricular infusion of OCN restored levels of enzymes needed for the biosynthesis of monoamine neurotransmitters and GABA. Additionally, symptoms of anxiety and depression could be relieved in these mice, while spatial learning and memory could be partially improved^7^. Furthermore, OCN levels decline with age and old mice perform worse in cognitive tasks than young mice. In line with these findings, intravenous injection of plasma from three-month old mice into 16-month-old mice elicited an improvement in cognitive performance and a decrease of anxiety-like behaviors in older mice – but only when the plasma contained OCN^9^. These studies suggest a potential therapeutic effect of exogenous OCN administration in terms of cognition, anxiety, and depression-like behavior. Regarding the presumed mode of action of classic antidepressant drugs that elevate available 5-HT and NE contents in the synaptic cleft^10^, OCN appears to work in an analogous way since it elevates levels of enzymes involved in monoamine synthesis^7^.

Human studies suggesting a link between OCN and depression have shown that OCN levels are decreased in depressive patients in comparison to control subjects^11–13^. This might be due to elevated cortisol levels in depressive patients^14–16^ as cortisol negatively regulates OCN production on a transcriptional basis^17–20^. Downregulation of OCN by cortisol therefore could lead to decreased monoamine neurotransmitter levels. This in turn means that OCN might interact with the hypothalamic-pituitary adrenal (HPA) axis which itself has been implicated in the pathogenesis of major depressive disorder^21,22^. While it remains to clarify to which extent OCN contributes to anxiety and depression and/or is involved in the pathogenesis of these disorders, the cited studies suggest that there is a clear link between OCN levels and proper functioning of the brain.

Based on the above-mentioned molecular and behavioral studies, we investigated the effects of intravenously administered OCN on the wild type mouse brain directly *in-vivo* via multiparametric magnetic resonance imaging (MRI). We used female mice in this study to ensure comparability, as previous publications investigated OCN’s effects on the brain in females^7,9,23^. Additionally, depression is more common in females as in males^24–26^. Since GPR37 and GPR158 were identified as OCN receptors in the mouse brain^9,27^, we generated an overview of the expression of the two corresponding genes using *in-situ* hybridization data of the Allen Brain Institute. Pharmacological MRI (phMRI) was conducted to examine the binding of OCN to specific functionally related brain regions. Those regions should partially overlap with the gene expression data. Resting state functional MRI (rs-fMRI) was performed to detect modulations in functional connectivity (FC) after OCN injection. Overall, we aimed to gain insight into the *in-vivo* effects of OCN in the female wild type mouse brain by integrating gene expression, phMRI and rs-fMRI. We identified four brain regions (brainstem, limbic output, association cortex, and basal ganglia) which play a major role in mediating OCN’s effect in the brain.

## Materials and methods

### Study design

**Fig. 1:**
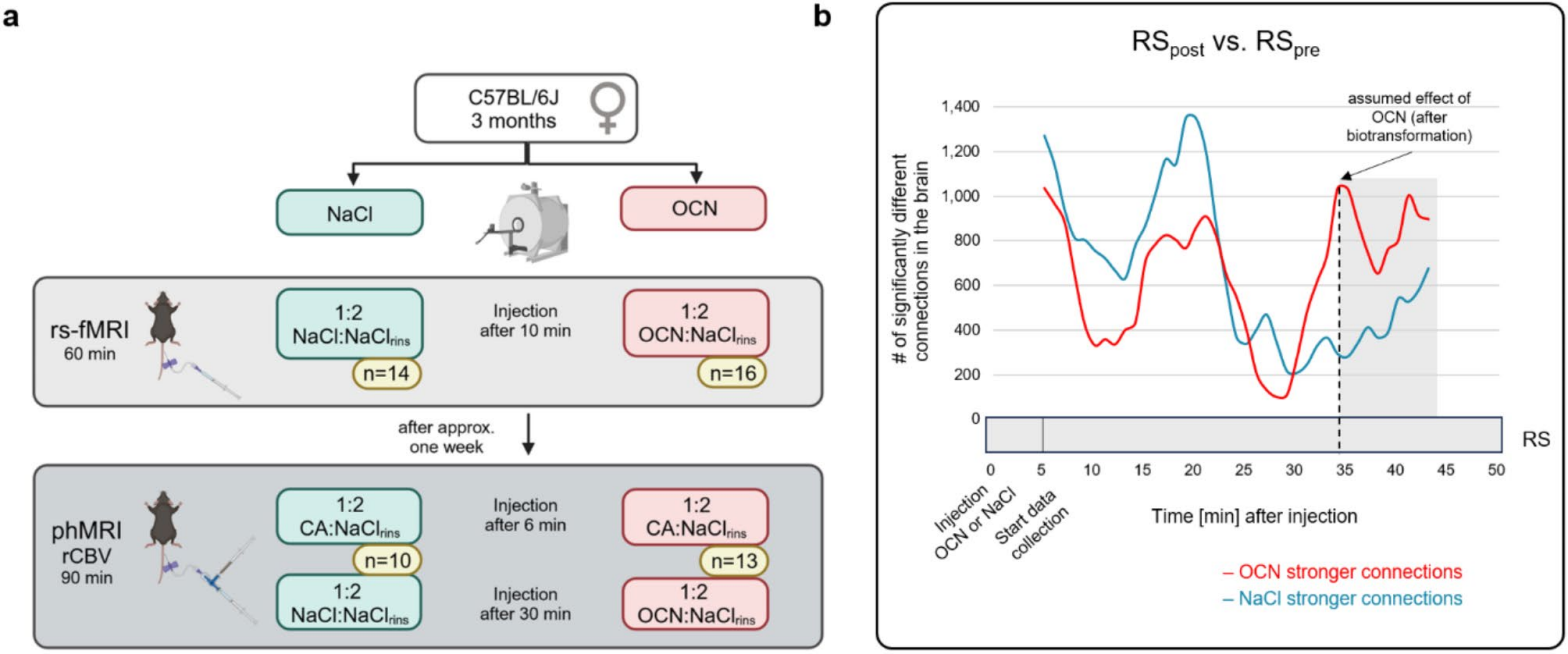
Study overview and time-dependent biotransformation effect of OCN. (**a**) The study consisted of a control group (injection with physiological sodium chloride; NaCl) and an OCN group (injection with OCN dissolved in NaCl). After each injection, NaCl for rinsing (NaCl_rins_) was applied. Each mouse in each group received first a rs-fMRI and second a phMRI measurement. For phMRI, contrast agent (CA) was applied prior to the respective substance. Figure was created with BioRender.com. (**b**) The graphs depict the components with their number (*p*_FWE_ < 0.05) of significantly different connections (*p* < 0.01) in the whole brain over the period of the RS_post_ measurement. For this, time windows of the RS_post_ measurement were tested statistically against the window of the RS_pre_ measurement of the respective group using NBS. At the time window of approx. 34 min after substance injection (dashed vertical line), the OCN group (red graph) showed the component with the highest number of significantly different connections (arrow). The duration of the time window (10 min) is symbolized by the light grey box.

The objective of this study was to examine the effects of intravenously injected OCN on the wild-type mouse brain by multiparametric MRI. Female C57BL/6J mice (n = 32) were randomly divided into a control group that received injections of sodium chloride (i.e., physiological NaCl) and into an OCN group that received injections of OCN solved in NaCl. Two mice of the NaCl group died after randomization and before the first rs-fMRI measurement which is why we included the remaining 30 as the total number of mice in our experiments. Each mouse underwent in total two MRI measurements with the respective substance injection (Fig. 1a). First, the rs-fMRI measurement was performed (n = 14 in the control group, n = 16 in the OCN group) to measure resting state (RS) connectivity, followed by the phMRI measurement after approx. one week which measures the relative cerebral blood volume (rCBV). In each experimental group, three mice died during the phMRI measurements and the phMRI measurement of one mouse was excluded in the control group due to strong artefacts in the imaging data resulting in n = 10 in the control group and n = 13 in the OCN group. Reasons for the time interval of one week between measurements were 1) to ensure enough recovery time for the mouse tail which facilitates the second intravenous injection for phMRI and 2) to ensure a complete degradation of the first injected OCN dose. OCN metabolism in mice is regulated by posttranslational O-glycosylation, and the *in-vivo* half-life of non-glycosylated OCN was shown to be approx. 108 min^28^. Since contrast agent is injected for phMRI measurements^29^, we conducted these experiments after the rs-fMRI measurement to exclude any imaging artefacts that could arise through possible contrast agent accumulation in the body of the mice. We chose to perform both rs-fMRI and phMRI in the very same animal to adhere to the Three R’s Rule^30^. After the experiments, we euthanized the animals with a CO_2_-overdose and subsequent cervical dislocation.

### Animals and housing

Female C57BL/6J mice (Charles River Laboratories, Sulzfeld, Germany) were housed in groups of 5 at the Institute of Pharmacology and Toxicology, Friedrich-Alexander University Erlangen-Nuremberg in a 12h-light–dark cycle at a temperature of 21 °C. Food and water were accessible ad libitum. Experiments were carried out during the light cycle on 3-month old mice and were approved beforehand by the local ethical committee for research animal care of the Regierung von Unterfranken. The experiments comply with the ARRIVE guidelines, the U.K. Animals (Scientific Procedures) Act from 1986 and were carried out in accordance with the European Communities Council Directive (2010/63/EU).

### Gene expression analysis

The database for *in-situ* hybridization whole-brain gene expression in the mouse brain, provided by the Allen Institute for Brain Science^31^, was used to assess expression patterns of the OCN receptor genes *Gpr158* and *Gpr37*. The 2D expression of these genes is registered into the Allen Brain Atlas (ABA) Common Coordinate Framework 3^32^ that comprises over 800 brain regions and is summarized in expression data grid volumes of 200 µm × 200 µm × 200 µm. In our study, these brain regions were further summarized into functionally related groups, which yielded 103 brain regions per hemisphere. The region-specific mean expression density of the genes was calculated by averaging the expression densities of all voxels belonging to the corresponding brain region. To visualize the mean expression density of both genes, the density values were color-coded and projected onto three exemplary ABA slices.

### rs-fMRI and phMRI preparation

An intravenous catheter was placed under isoflurane anesthesia in the lateral tail vein of the mice randomly on the left or right side. The mice were placed on an acrylic cradle with an integrated water heating system. The head of the mice was fixed in an anatomical nose mask by the teeth to minimize possible movement of the mice’s head during MRI measurements. Isoflurane anesthesia was supplied constantly through this mask (0.8–1.3%) during experiments. A pediatric breathing sensor (Graseby® Respiration Sensor, Smiths medical, Inc., Minneapolis, MN, USA), placed under the mice’s chest, constantly monitored the breathing rate. The eyes were covered with eye ointment (Bepanthen, Bayer Vital GmbH, Leverkusen, Germany) to prevent them from desiccation. The catheter was extended with an extension hose containing the injection substances for the respective MRI measurement. The extension hose was filled for rs-fMRI measurements (Fig. 1a) with a 1:2 ratio of carboxylated OCN (90 ng/50 µl per mouse, solved in NaCl, MyBiosource, Inc., San Diego, USA) to NaCl for rinsing (NaCl_rins_) for the OCN group. For the NaCl group, the hose was filled 1:2 with NaCl:NaCl_rins_ (0.9%, BRAUN, Melsungen, Germany). For phMRI (Fig. 1a), the extension hose was filled via a three-way valve (Discofix®, BRAUN, Melsungen, Germany) to inject 1:2 iron-based contrast agent (15 mg/kg; FeraSpin™ R, Viscover™, Berlin, Germany) to NaCl_rins_ followed by 1:2 OCN:NaCl_rins_ (OCN group) or 1:2 NaCl:NaCl_rins_ (control group).

### MRI scanner parameters

#### rs-fMRI and phMRI scan preparations

Mice were positioned in a 4.7 Tesla Bruker Biospec 47/40 with a 40 cm bore and 200 mT/m gradient coil system (Bruker Biospin MRI GmbH, Ettlingen, Germany). An anatomically shaped 3 cm 4-channel array head coil (RAPID Biomedical GmbH, Rimpar, Germany) was used for signal detection. Excitation took place via an actively decoupled RF-coil-system. Potential head motion of the mice in the scanner was assessed using echo planar imaging (EPI; TEeff = 25 ms, TR = 200 ms, matrix of 64 × 64 voxel, FOV 15 mm × 15 mm, 0.5 mm slice thickness, one slice, in-plane resolution of 0.234 mm × 0.234 mm) and the mounting and fixation of the mice was corrected if the head movement was larger than one pixel to the initial position. A rapid acquisition relaxation enhancement (RARE) sequence (TEeff = 56 ms, TR = 2669 ms, matrix of 128 × 128 voxel, FOV 15 mm × 15 mm, 0.5 mm slice thickness, 16 coronal slices, k-space averaging 4; RARE Factor = 8) was recorded to obtain an anatomical reference for the fine-positioning of the functional volume. For this, the 10^th^ slice (from the anterior side) was positioned at the distal end of the lateral ventricle of the reference and the angle of the slices was adjusted to the position of the mice’s head in the scanner. The following functional sequences were conducted based on this adapted slice geometry and position. The final volume spanned the brain from Bregma -8.00 mm to 2.46 mm.

#### rs-fMRI measurement

The rs-fMRI measurements were performed for each mouse by first recording a 10 min RS baseline (RS_pre_) by using EPI sequences of 22 axial slices (TEeff = 25 ms, TR = 2000 ms, matrix of 64 × 64 voxel, FOV = 15 mm × 15 mm, slice thickness 0.5 mm, in-plane resolution of 0.234 mm × 0.234 mm, 300 repetitions). After the RS baseline scan, the respective substance (OCN or NaCl) was immediately injected via the extension hose, followed by a 5 min rest period to allow for substance distribution (Fig. 1a,b). After the rest period, 50 min of RS after injection (RS_post_) were recorded (same scan parameters as for RS_pre_ but with 1500 repetitions) to monitor the changes in FC during substance influence.

#### phMRI: rCBV measurement

The phMRI measurements were performed using fast low angle shot (FLASH) MRI (TEeff = 10 ms, TR = 625 ms, matrix of 128 × 128 voxel, FOV = 15 mm × 15 mm, slice thickness 0.5 mm, in-plane resolution of 0.117 mm × 0.117 mm, 90 repetitions, total duration of 90 min). 6 min after the start of the measurement, iron-based contrast agent was injected via the extension hose (as described above, cf. Fig. 1a), followed by the respective substance (OCN or NaCl) injection at 30 min recording time (24 min after contrast agent injection). The recording then continued for 60 min to evaluate changes in rCBV.

### Data analysis

#### Preprocessing

Data was preprocessed by correcting for inter-slice scan time (ascending interleaved, interpolation method cubic spline) and motion (registration to first brain volume, trilinear detection and sinc interpolation) in BrainVoyager (for details, see *Software*). To assess motion, the raw data of every animal was evaluated calculating framewise displacement (FD)^33^ (6 motion parameters) to detect FD outliers (exceeding 0.2 mm displacement). If FD outliers > 5% existed, they were checked for successful motion correction using standardized derivative of time courses, variance across spaces (zDVARS)^34,35^. zDVARS in the range of 1.0 ± 0.1 was considered as good motion correction. All animals met the inclusion criteria of both FD and zDVARS. After quality assessment, brain masks were drawn manually for each animal to segment the brain. The first volume of each scan was registered slice-wise semi-automatically to a high-resolution anatomical template (adapted from the ABA) via grey value-based affine registration (4 degrees of freedom; x and y translation, x–y isotropic scaling, z rotate). Rotation was adjusted using one middle slice and the resulting rotation was applied to all slices to prevent twisting of the brain. Shifting in z was not necessary, as exact positioning of the slices of all mice was ensured during measurements. This procedure leads to better results than a fully automated volume-based workflow for the registration of functional images (for registered mean of both groups, see Supplementary Fig. S1). An in-house created mouse brain atlas (comprising 206 brain regions, adapted from the ABA^32^) was matched manually to the mean of the registered volumes of all animals so that anatomical features corresponded resulting in regions of interest (ROI) for both hemispheres. This matched brain atlas was inversely registered by using the registration matrix of every individual animal, yielding subject-specific ‘label masks’. The quality of the inverse registration was assessed visually and corrected if not satisfactory.

#### Resting state analysis

RS data was further preprocessed using 3D Gaussian smoothing with FWHM 0.7 mm, lowpass filter 0.1 Hz and removal of global signal mean by linear regression. For conducting multi-seed region analysis (MSRA)^36^, seeds consisting of five voxels were automatically placed in the center of gravity of each brain region and their mean time courses were correlated (Pearson’s r) with the time course of each voxel in the brain. This resulted in one correlation map per seed. These correlation maps were thresholded using FDR according to Benjamini-Yekutieli (BY-FDR)^37^ to account for multiple comparisons and extract significantly correlating voxels. This more conservative version of the FDR considers dependencies between neighboring voxels. Mean correlation values per brain region of one thresholded seed correlation map were calculated (via transformation to Fisher’s z and back to Pearson r) and this procedure was repeated for each seed in each brain region resulting in asymmetric correlation matrices containing one correlation value per brain region (for details, see Kreitz et al.^36^). These correlation matrices were used to create network graphs consisting of nodes (brain regions) and edges (connections between brain regions).

As brain connectivity is not static over time^38^, we divided the RS_post_ measurement into time windows of 10 min (300 time points) each, shifted by one minute along the time course of the measurement, yielding 39 time windows over the period of 50 min (for visual explanation, see Supplementary Fig. S3). As the duration of the RS_pre_ measurement was 10 min, it consisted of only one time window. In order to identify the time window of the OCN effect, we used NBS (for details, see *Statistics*) to determine the highest number of connections that are significantly stronger in the respective time window compared to baseline (RS_post_ vs. RS_pre_). As the RS_post_ measurement of the OCN group showed the highest number of significantly different connections in the time window at 34 min after injection (Fig. 1b, dashed vertical line), this time window was considered for further analysis.

#### Graph-theoretical network assessment

Information flow within the brain, represented by network graphs, should differ clearly from random networks. This property is characterized by the global parameter small-world index^39^ which describes networks by the ratio between two key network metrics: 1) the average path length between all possible pairs of nodes in a graph and 2) the clustering coefficient which provides information about the fraction of a node’s neighbors that are neighbors of each other, denoting the level of connectivity. Both metrics are normalized by comparison with values of random networks with equivalent size. We calculated the small-world index over a density range of 2–20% of all possible connections for the RS baseline measurement of all animals, since the index depends on the number of edges between nodes, i.e. the network density. The resulting curve is a hyperbola (Supplementary Fig. S2) and the maximum curvature represents the maximal level of connectivity and sparsity which allows for a clear distinction from random networks. The maximum curvature was calculated where the first derivative of the normalized hyperbola is -1, which resulted in a density of 7% in our study. Consequently, further analyses for both groups were performed with a network density of 7%. Mean correlation matrices of both groups before and after density thresholding are shown in Supplementary Fig. S4. A unified density is important as it allows a topological comparison of brain networks across groups. It is of note that the direction of the graphs was determined through a data-driven approach, focusing on the correlation of target voxels, rather than representing a directed flow of information (therefore, pseudo-directed).

In a network, the hub score describes the relative quantity of information that is distributed from a given node and the authority score (both calculated with the hyperlink-induced topic search (HITS) algorithm) describes the relative quantity of received information by a given node^40,41^. After thresholding the brain networks of all animals at the density of 7% (see above), the hub score was determined for each brain region at the time window of 34 min after injection (i.e., time point of interest, cf. Figure 1b). Since the networks are solely pseudo-directed, the hub and authority scores were averaged per node and denoted as hub score (unweighted analysis).

#### rCBV analysis

Data was preprocessed as described above and analyzed as described in Wank et al. (2024)^42^. The rCBV data was smoothed in space (Gauss, FWHM 2.0 voxel) and time (polynomic Savitzky-Golay, filter width 17, using a polynomial fit with degree 16). Time smoothing started at 6 min, i.e. after the time point of contrast agent injection. The equilibration time for the contrast agent was set to 9 min, i.e. after 15 min, equal distribution of the contrast agent was assumed in the body of the mice. Therefore, the period of 15–30 min composed the baseline, which was used as the basis for exponential fitting to extrapolate the washout behavior of the contrast agent over the time of the recording. At 30 min, the injection of the respective substance (NaCl or OCN) took place during the phMRI measurement, and the period from 35 min (i.e., 5 min after injection) until 80 min was used for data analysis. To achieve similar time description between RS and rCBV, we describe this period in the following as 5–50 min after injection (for visual explanation, see Supplementary Fig. 3). The period of 5–50 min after injection was considered for data analysis, as a response of the injected OCN was expected in this period (Fig. 1b) and because the real washout kinetics may differ from the exponentially fitted one toward the end of the experiment, which would influence the calculated rCBV signal^42^. rCBV time courses were calculated relative to the fitted baseline for each time point (see Supplementary Fig. S5 for mean raw and rCBV curves of exemplary brain regions). The resulting rCBV data was detrended and processed further by smoothing temporally (running average, filter width 5) and spatially (median 3D filter, kernel size of 5 × 5 × 3 voxels). The AUC of the rCBV signal was calculated over the average time course for each animal from 5–50 min and averaged per group. The rCBV data was quantified by calculating maximum (peak) rCBV values and their corresponding time points for each voxel in each brain region and for each animal. Only voxels having a peak value in the period from 5–50 min were considered (see above).

#### Statistics

Due to unbalanced group sizes, ANOVA with regression was applied to test peak rCBV values (values > 0) and hub score with group and brain region as between subject factors. Follow-up t-tests were corrected for multiple comparisons using FDR according to Benjamini-Hochberg^43^. We used NBS as introduced by Zalesky et al.^44^ to delineate differences in network connectivity strength after injection in each group as well as between groups. Rather than analyzing individual connections independently, NBS focuses on identifying coherent groups of connections, or ‘components’ that exhibit significant differences between RS_post_ and RS_pre_ of the respective group as well as between the RS_post_ measurements of both groups. The method performs paired two-tailed t-tests (since the RS_pre_ measurement served as a baseline) on each connection in the network (*p* < 0.01) or homoscedastic t-tests between groups, identifying the largest component of coherent connections that are significantly different. These components are evaluated based on their size, defined by the number of connections they contain. NBS then applies permutation testing to assess the statistical significance of the observed component size, comparing it against a null distribution generated through 10,000 random permutations (random assignment of animals to groups for homoscedastic tests and random sign switch for paired tests). This process yields a family-wise error (FWE) corrected *p*-value (*p*_FWE_ < 0.05), representing the probability that the observed component size is not random.

#### Software

The scanner was operated with ParaVision 7.0.0 (Bruker Biospin MRI GmbH, Ettlingen, Germany). MRI data was preprocessed (motion correction and inter-slice scan time correction) in BrainVoyager QX (Brain Innovation, Maastricht, Netherlands; version 2.8.2.2523). Amira (Zuse Institute Berlin, Berlin, Germany and Thermo Fisher Scientific, Waltham, USA, version 5.4.2) was used for visualization of the color-coded gene expression patterns and of the network graphs. RS and rCBV data were analyzed using MagnAn (BioCom GbR, Uttenreuth in IDL, Excelis Visual Information Solutions, Inc., a subsidiary of Harris Corporation, Melbourne, USA, version 2.5.13). To provide a whole brain overview and additional regional information, data were displayed using BrainWheel (V1.0), an in-house created extension of the circlize package^45^ in RStudio (Posit PBC, Boston, Massachusetts, USA, version 2023.12.1).

## Results

### *Gpr158* is highly expressed in the cortex and basal ganglia whereas *Gpr37* shows high gene expression in thalamus and brainstem

**Fig. 2:**
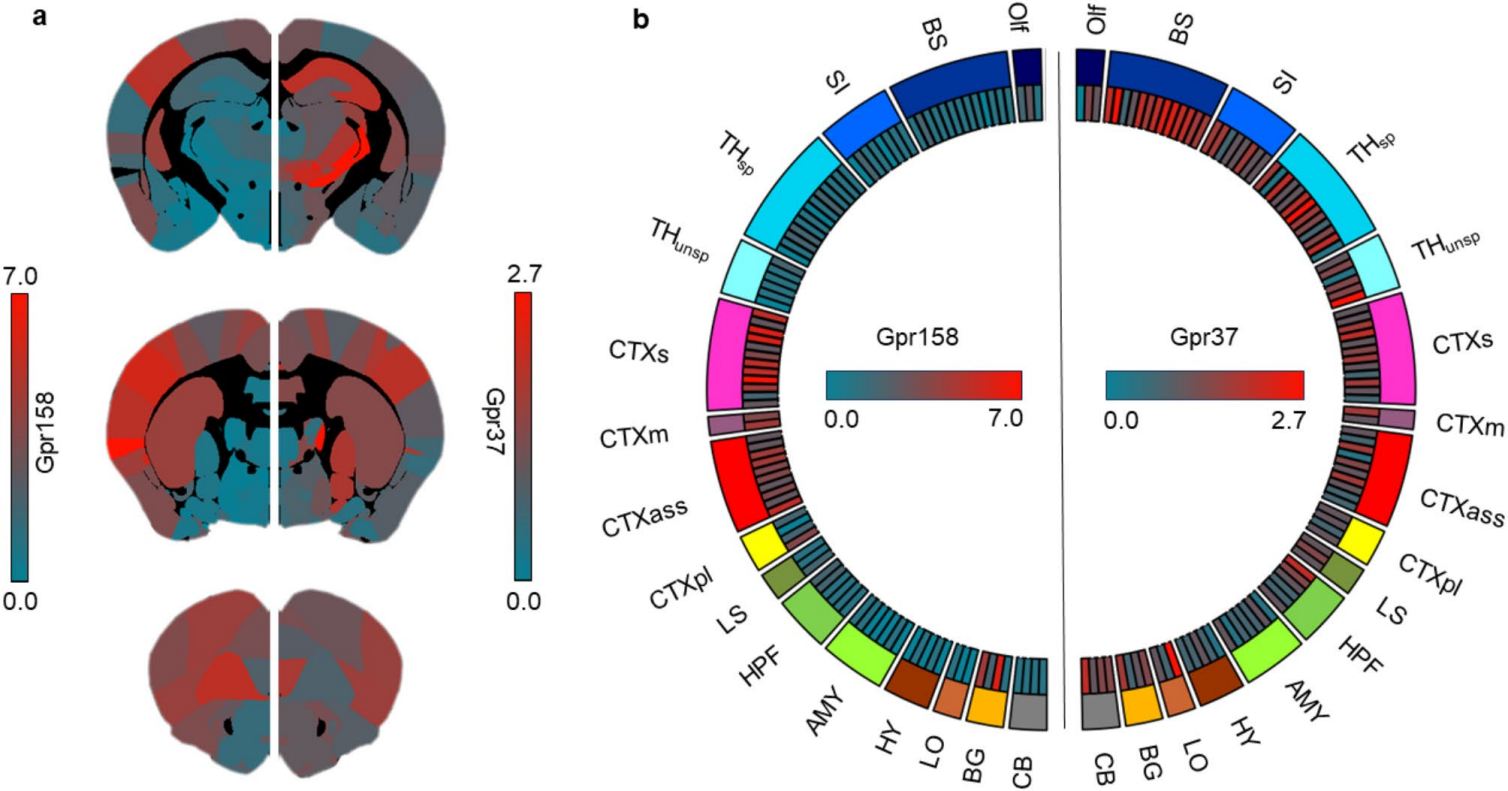
Gene expression of *Gpr158* and *Gpr37* in the mouse brain. (**a**) The mean gene expression densities for genes *Gpr158* (left) and *Gpr37* (right) are displayed anatomically on three exemplary coronal slices from the Allen Brain Atlas. Densities are color-coded for low mean expression densities being blue colors to high mean expression values being red colors. Note the different ranges of the mean expression densities of both genes. (**b**) The circular representation of gene expression shows the color-coded mean expression density (inner circle) with the assignment to functionally related groups (outer circle) for *Gpr158* on the left side and *Gpr37* on the right side. Note that the mean expression densities are displayed without left and right information due to the gene expression data provided by the Allen Brain Institute, as it only includes information for one hemisphere for *Gpr158*. Abbreviations of the corresponding brain regions, their assignment to functionally related groups and the exact values of the respective mean expression densities can be found in the Supplementary Table S1.

Gene expression analysis of *Gpr158* and *Gpr37* revealed different expression patterns across the brain. *Gpr158* has a dominant expression density in sensory, motor, association, and paralimbic cortex as well as in the basal ganglia (Fig. 2). *Gpr37* is highly expressed in thalamus, brainstem, in the limbic output as well as in in the hippocampal formation, sensory and motor cortex (Fig. 2; for assignment of brain regions to their functionally related groups see Supplementary Table S1). It is noteworthy that the maximum mean expression density for *Gpr158* reaches up to around 7, whereas maximum values for *Gpr37* are around 2.7. A detailed list of the brain regions with their respective mean expression density for each gene can be found in Supplementary Table S1.

### OCN elicits region-specific changes in rCBV with significant increases in subcortical and limbic blood volume

**Fig. 3:**
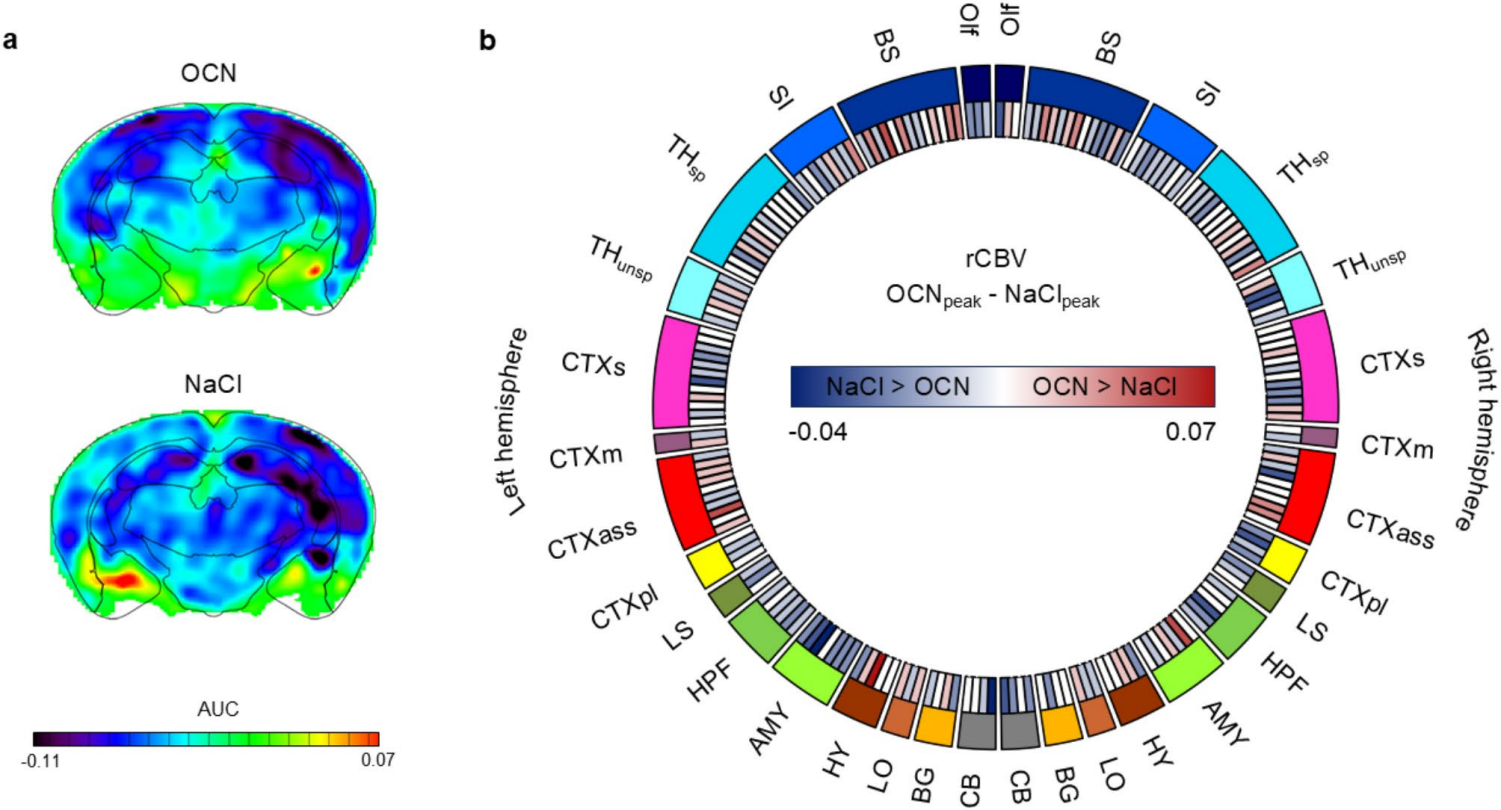
Direct target regions of OCN in the brain assessed via regional cerebral blood volume. (**a**) AUC of rCBV signals of OCN (top) and control group (bottom) between 5–50 min after injection represented on one exemplary slice (Bregma -2.06 mm). Corresponding brain region contours are overlaid to gain anatomical insight into the signal pattern. (**b**) Circular representation of the difference of the average peak rCBV values of OCN and NaCl group per brain region for left and right hemisphere. Abbreviations of the corresponding brain regions, their assignment to functionally related groups, and the exact values for the rCBV differences can be found in Supplementary Table S2.

The rCBV signal reflects local increases or decreases in blood volume that occur due to changes in metabolic demand of distinct brain regions. Those changes can arise due to e.g., ligand-receptor binding. In phMRI measurements, the OCN group showed more positive rCBV signals compared to the NaCl group in thalamus, hypothalamus, regions of the amygdala, and paralimbic cortex (Fig. 3a). The average rCBV peak values differed significantly between both groups (ANOVA, *p* = 0.00005; df = 1). The time point at which the highest number of voxels peaked in their rCBV signal occurred in the OCN group at approx. 37 min after injection. Although ANOVA did not reveal significant interaction, we investigated region-specific differences to see a tendency through which the main effect is triggered. The difference between the average peak rCBV values of the OCN group and the average peak rCBV values of the NaCl group (Fig. 3b) elicited that nearly 68% of all brain regions showed stronger signals whereas only around 29% of brain regions showed weaker signals in the OCN than in the NaCl group. This suggests that OCN injection might be associated with a region-specific increase in blood volume, potentially at specific time points during the measurement. Regions influenced by OCN injection, and therefore regions in which OCN potentially binds to its receptors and changes the metabolic demand of the respective brain regions, were brainstem, association cortex, amygdala, and regions of the thalamus and hypothalamus, among others. For the exact differences of average peak values of the brain regions that are displayed in Fig. 3b, see Supplementary Table S2.

### OCN induces widespread network connectivity modulations

**Fig. 4:**
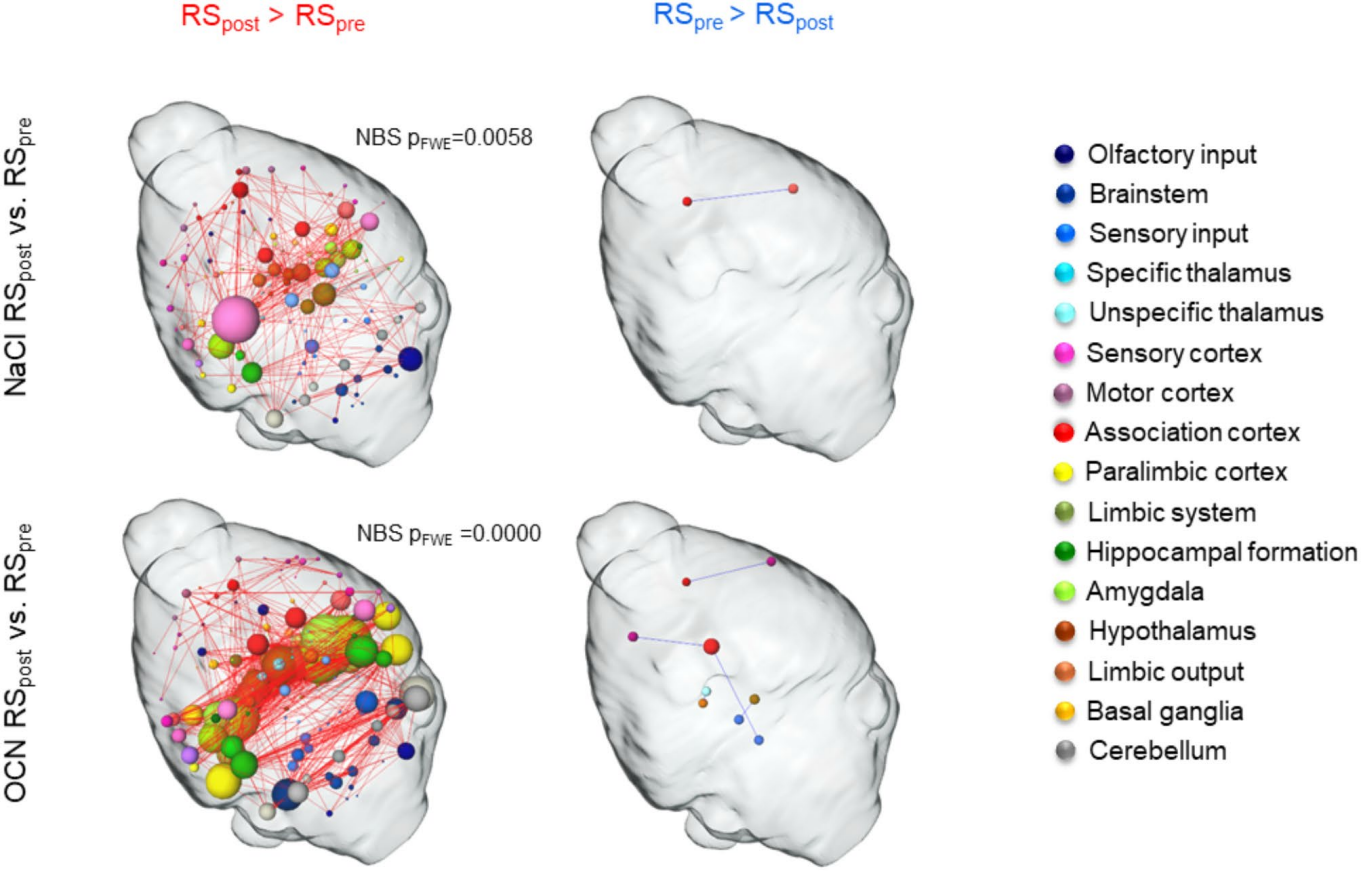
Time-dependent modulation of functional connectivity 34 min after injection compared to baseline. Significant changes in modulated connectivity 34 min after injection of NaCl (top) and OCN (bottom) compared to baseline (RS_pre_), calculated with NBS (*p*_FWE_ < 0.05, *p* < 0.01). Left networks show components that were significantly stronger after injection (red edges), right networks show components that were significantly stronger before the respective injection (blue edges). Networks are composed of color-coded nodes (see legend) representing brain regions and edges representing the connections between those regions. The size of the nodes correlates with their degree, i.e. the number of connections they possess.

The resting state (RS) describes the activity of the brain at “rest” when it does not receive a sensory or cognitive stimulus^46^. While rCBV mainly indicates directly targeted regions of OCN, RS can give insight into secondarily modulated regions and changes in network connectivity. Since RS functional connectivity (FC) and the effect of the substance injection is dynamic over time, we applied sliding window analysis (see Materials and Methods). The network-based statistics (NBS) component (i.e., coherent group of connections, see Materials and Methods) with the highest number of significantly different connections occurred at 34 min after OCN injection (Fig. 1b). This time window with a duration from 34 to 44 min showed over 1,000 significantly stronger connections after vs. before injection in the OCN group, whereas the NaCl group showed less than 400 significantly stronger connections which reflects the undisturbed dynamic over time. We therefore assumed that this time window might be the crucial one to delineate OCN’s effect on the brain. The RS component in this time window (Fig. 4, left) showed that OCN modulated more brain regions than NaCl. The number of significantly modulated connections was also higher in the OCN group compared to control. Among others, the amygdala, hypothalamus, cerebellum, hippocampal formation, paralimbic cortex, brainstem, and association cortex showed the highest number of modulated connections (in descending order). Only few connections were found to be stronger before the injection of the respective substance (Fig. 4, right).

### Amygdala, hypothalamus, and the limbic output emerge as key hubs after OCN administration

**Fig. 5:**
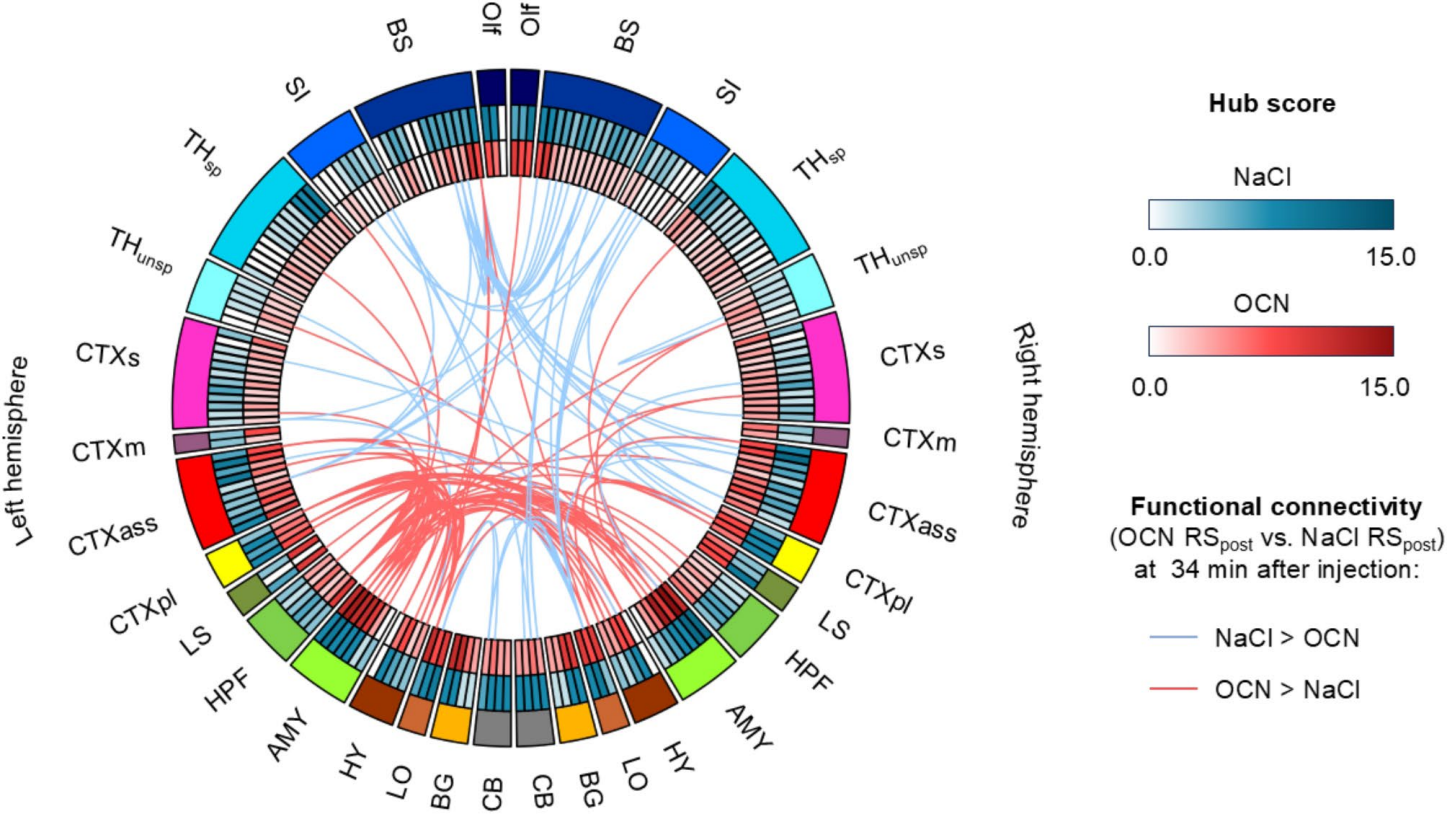
Circular representation of hub scores and the functional connectivity of OCN vs. NaCl group at 34 min after substance injection. The hub scores (normalized, sum of all hub scores is 100) for both hemispheres at 34 min after injection are displayed for both groups. The modulated functional connectivity of OCN vs. NaCl group calculated via NBS (p_FWE_ < 0.05, p < 0.01) is displayed in the center of the circular plot. Abbreviations of the corresponding brain regions, their assignment to functionally related groups, and the exact values for the hub scores can be found in Supplementary Table S2.

Hubs are key brain regions particularly important for the efficient transfer of information within the brain. They are essential for the proper functioning of the brain and their removal would lead to major distortions in the transmission of information in the network. Nuclei of the amygdala had the highest hub score in the OCN group, followed by brain regions belonging to basal ganglia, limbic output, the limbic system, brainstem, association cortex, and the hippocampal formation (Fig. 5). ANOVA elicited a significant interaction (*p* < 0.0001, df = 205), indicating that the injection (NaCl vs. OCN) has differential effects on the brain regions. Follow-up t-tests showed that the above-mentioned brain regions showed significant differences only before correction for multiple comparisons, therefore indicating only a trend toward regions-specific effects. For exact hub scores of the brain regions of both groups, see Supplementary Table S2. Next, we directly compared the RS FC between OCN and NaCl for the time window at 34 min, again using NBS (Fig. 5). The analysis showed that some of the connections that were stronger in the OCN group were connections between brain regions that were also classified as important hubs, such as the amygdala, the hypothalamus, the limbic output, paralimbic cortex and the hippocampal formation.

### OCN modulates rCBV and functional connectivity in the mouse brain, targeting specific brain regions with high *Gpr158* and *Gpr37* expression

**Fig. 6:**
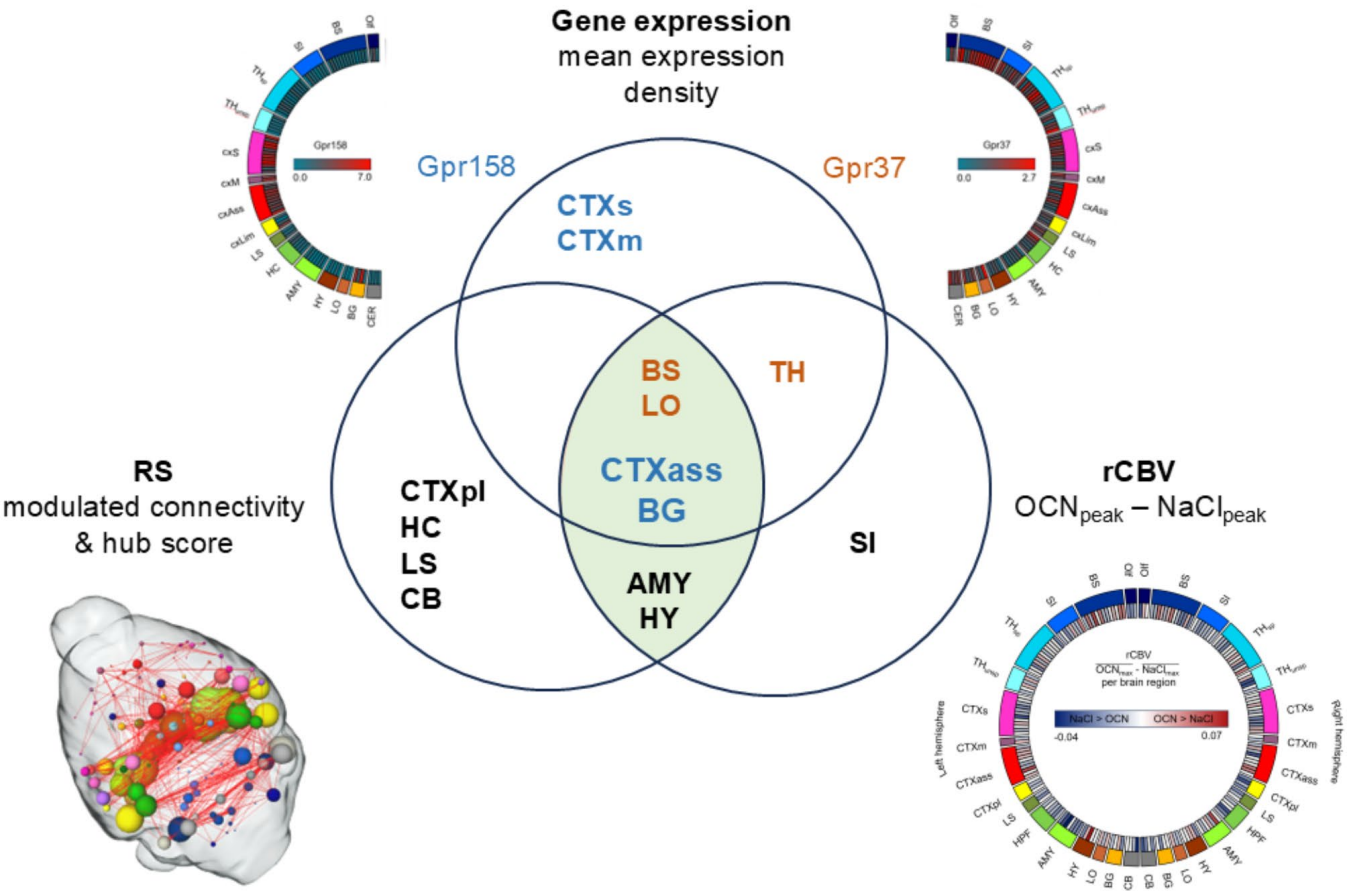
Summary of the top 10% modulated brain regions due to OCN administration revealed by multiparametric MRI and gene expression. The three overlapping circles contain brain regions that are in the top 10% regarding their respective largest values for gene expression (upper circle), difference of average peak rCBV values of OCN minus NaCl group (right circle), as well as hub score and modulated connectivity (left circle) of the OCN group. Brain regions in intersections indicate common results in two or all analytical modalities. Areas highlighted in green indicate overlap of MR modalities. To separate additionally between genes, brain regions written in blue highly express *Gpr158*, brain regions written in brown highly express *Gpr37*. Regions written in black do not belong to the top 10% of either *Gpr158* or *Gpr37*. Abbr.: CTXs: sensory cortex, CTXm: motor cortex, CTXpl: paralimbic cortex, HC: hippocampus, LS: limbic system, CB: cerebellum, BS: brainstem, LO: limbic output, CTXass: association cortex, BG: basal ganglia, AMY: amygdala, HY: hypothalamus, TH: thalamus, SI: sensory input.

The present study showed that intravenously administered OCN in female wild-type mice elicited a clear effect in the brain in terms of change in rCBV and FC. Our analysis of gene expression data from the Allen Brain Institute provided potential target regions of OCN, and *in-vivo* multiparametric MRI confirmed OCN’s action on several of those regions. Figure 6 provides an overview of the top 10% of all analyzed brain regions regarding their values for gene expression, hub score, modulated connectivity, and difference of average peak rCBV signal. Association cortex, basal ganglia (both highly expressing *Gpr158*), brainstem, and the limbic output (both highly expressing *Gpr37*) were found to be in the top 10% of all three analytical modalities. The thalamus expressing *Gpr37* matched the rCBV data; hypothalamus and amygdala were highly relevant in RS and rCBV but do not show notable expression of either *Gpr158* or *Gpr37*. All other regions in the top 10% (sensory and motor cortex, paralimbic cortex, hippocampus, limbic system, cerebellum, and sensory input) showed no intersections with the respective other analytical modalities.

## Discussion

The background promoting the detailed study of OCN’s action in the brain is its involvement in monoamine neurotransmitter regulation in the brain, its interaction with the HPA axis as well as the evidence from previous animal studies in which OCN^-/-^ mice exhibited cognitive deficits, increased anxiety and depression-like behavior. To take a step further in understanding OCN’s physiological role, we provide first *in-vivo* imaging data on the effects of OCN on the healthy brain of mice using multiparametric MRI.

We identified four brain regions that play an important role in all our analytical modalities: brainstem and limbic output which express high levels of *Gpr37* and the association cortex and the basal ganglia which highly express *Gpr158*. PhMRI indicates direct effects of OCN, whereas rs-fMRI indicates secondarily influenced brain regions. As the above-mentioned brain regions overlap in all three modalities, these are likely the core brain regions that respond to OCN. Amygdala and hypothalamus, which do not show high expression of the two genes, are likely modulated by those core regions. This might be e.g., because these brain regions are essential for mediating the effect of OCN. The thalamus showed high expression of *Gpr37* and was targeted by OCN which corresponds to its important role as “gateway” to the cortex. Thalamic circuits mediate sensory input to the cortex (first-order relay)^47^ or input from the cortex resulting in output to different brain regions, e.g., the basal ganglia (second-order relay)^47,48^. OCN’s action through receptor GPR37 was shown to play a significant role in myelin homeostasis in the central nervous system, including the differentiation and maturation of oligodendrocytes, mediated by the key transcription factor *Myrf*^27^*.* This implicates that distortions in this system affect the proper functioning of neurons such as saltatory nerve conduction^27^, which in turn can lead to impairments in neuronal survival, regulation of energy demand and motor skills, ultimately contributing to distortions of higher brain function and neuropsychiatric diseases^49^.

From the thalamus, different subnuclei project to structures such as hippocampus and medial prefrontal cortex (cognitive functions; CTXass in our subdivision), as well as amygdala and nucleus accumbens (basal ganglia in our subdivision). Those brain regions are involved in affective behaviors such as stress and anxiety^48^. Amygdala, hypothalamus, and brainstem nuclei are connected in a network of stress-related neurocircuits and the hypothalamus itself is connected to the HPA axis as well as to neurotransmitter and neuropeptide regulation^50^. Subregions of the limbic output such as the periaqueductal gray^51^ were shown to be associated with depression, and the zona incerta is seen as an integrative node for global behavioral modulation, e.g., regulating defensive behavior or conveying motivational drive^52^. Core regions influenced by OCN in our study as well as the brain regions occurring in two of three modalities therefore, can be associated with stress, anxiety, and depression. On a molecular basis, decreased levels of brain-derived neurotrophic factor (BDNF), a neurotrophin responsible for survival and differentiation of neurons^53^, were found in patients with major depressive disorder^54^. The hippocampus and the prefrontal cortex are primarily affected^55^ and antidepressant treatment leads to an up-regulation of BDNF^56^. Since OCN signaling through GPR158 is mediated via BDNF in the hippocampus^9^, this connects OCN to depression, but also to learning and memory, as shown in OCN^-/-^ mice that performed worse in the Morris Water Maze compared to control^7^. The hippocampus is the primary region associated with learning and memory^57^ and the rhinal cortex (CTXpl) is closely interconnected with the hippocampus^58^. Both hippocampus and rhinal cortex interact in object recognition^59,60^ which is important for the Morris Water Maze task, as mice have to learn to find the submerged platform using spatial cues. Hippocampus and the paralimbic cortex are included in our top 10% regions in RS, including them as secondarily modulated regions.

Our data showed slight asymmetric effects of OCN in the brain (e.g., in Fig. 3). This is consistent with previous rCBV studies^61^ and is suggested to be related to the natural lateralization of the mammalian brain. Physiological brain lateralization is observed, e.g., in brain structure^62^ and neurotransmitter distribution^63^. Receptor and gene distributions are also known to occur asymmetrically in the brain^64^. Since our gene expression data covered only one hemisphere, we cannot determine whether *Gpr158* and *Gpr37* show lateralized expression. However, this remains a possibility that could explain the observed rCBV asymmetry.

To our best knowledge, there is no data available on the duration of biotransformation of the carboxylated to the undercarboxylated form of OCN in mice and its pharmacokinetics regarding its transfer across the BBB. We determined a time window at 34 min after injection of OCN, in which the animals showed the component with the highest number of modulated, significantly different connections. We therefore assumed that this time window might represent the time shortly after biotransformation of OCN, in which it started to be present in its decarboxylated, bioactive form that can cross the BBB and act on the brain. As the first peak of significantly different connections between 18 and 20 min occurred in both groups (Fig. 1b), this might be due to the reaction of the brain to the blood volume change after substance injection. The second peak at 34 min occurred only in the OCN group which is why we attributed this peak to the actual effect OCN exerts in the brain. Hence, sufficient biotransformation of the OCN bolus and the passage across the BBB would occur shortly before 34 min, as its effect on the brain requires the decarboxylated form. This finding emphasizes the utility of the sliding-window method, as different periods after injection can be assessed separately and highlight OCN’s time-dependent effects on the brain.

All these findings are based on female physiology which poses a limitation to this study. As OCN was found to influence positively male but not female fertility^2,65^, and a decrease in testosterone was found to be associated with depression and anxiety^66,67^, male mice could be affected differently by OCN injection or knockout. This supports the investigation of female mice first to detect the “pure” effect of OCN on the brain, as influences from sex hormone regulation are minimized.

As we used wild-type mice in our study that naturally produce OCN, we cannot account for basal effects of physiological OCN production, however, as we imaged mice directly after high-dosed OCN injection, the basal effect possibly contributing to our results should be minimal.

In regards of the gene expression data from the Allen Brain Atlas (ABA) database presented in our work, it is important to keep in mind that these data represent 1) male C57BL/6J gene expression and 2) only a snapshot of existing average gene expression in healthy mice which ignores individual variability. We performed the experiments using female C57BL/6J mice, which minimizes genetic variability, though sex-specific differences in gene expression could impact the results.

Our sliding-window analysis suggests the time window of 34–44 min for the effect of OCN and the time point of the strongest rCBV response at 37 min corroborates this suggestion. Hence, biotransformation must take place earlier to enable the crossing of the hormone across the BBB.

All animals in the OCN and NaCl group were anesthetized during both RS and rCBV measurements. This ensures the minimization of the mice’s movement during measurements and provides comparability between animals and groups. Moreover, anesthetized mice are not exposed to the stress mice would experience during the scanning process in a wake state. Several studies examined the effects of isoflurane which might influence, e.g., the cardiovascular system or spontaneous neural activity^68,69^. Studies investigating functional connectivity in awake vs. anesthetized rodents showed that low anesthetic doses (e.g., 1% isoflurane) maintain the connectivity pattern^70^. As isoflurane doses in this work were kept at approx. 1%, a major confounder through anesthesia can be excluded.

## Conclusion

We used multiparametric MRI to identify brain regions influenced by OCN through direct effects and secondary modulation in the wild type female mouse brain and integrated these findings with gene expression data for the two known central OCN receptors, GPR158 and GPR37. This approach revealed specific regions with high expression of these genes that are relevant to RS connectivity, rCBV, or both, enhancing our understanding of OCN’s central physiological effects. Brain regions influenced by OCN are associated with learning and memory, as well as anxiety and depression-like behavior. These insights contribute to the establishment of OCN’s role in the pathogenesis of anxiety and depression, and to the investigation of whether OCN may prove to be a future therapeutic agent for these disorders.

## Electronic supplementary material

Below is the link to the electronic supplementary material.

Supplementary Material 1

## Data Availability

The data that support the findings of this study are available from the corresponding author upon reasonable request.
