# Supplementary material for: Pharmacological and resting state fMRI reveal Osteocalcin’s effects on mouse brain regions with high Gpr37 and Gpr158 expression
Source: Sci Rep. 2025 Mar 24;15:10116. doi: 10.1038/s41598-025-95000-2 (PMC11933355; doi:10.1038/s41598-025-95000-2)
Supplement: Supplementary file 1 — Supplementary Material 1 [file 41598_2025_95000_MOESM1_ESM.pdf]

Supplementary Information

Pharmacological and Resting State fMRI Reveal Osteocalcin's Effects on Mouse Brain Regions with High Gpr37 and Gpr158 Expression

Natalia K. Freus, Isabel Wank, Maximilian Häfele, Liubov S. Kalinichenko, Christian P. Müller, Sandra Strobelt, Andreas Ludwig, Andreas Hess, Silke Kreitz

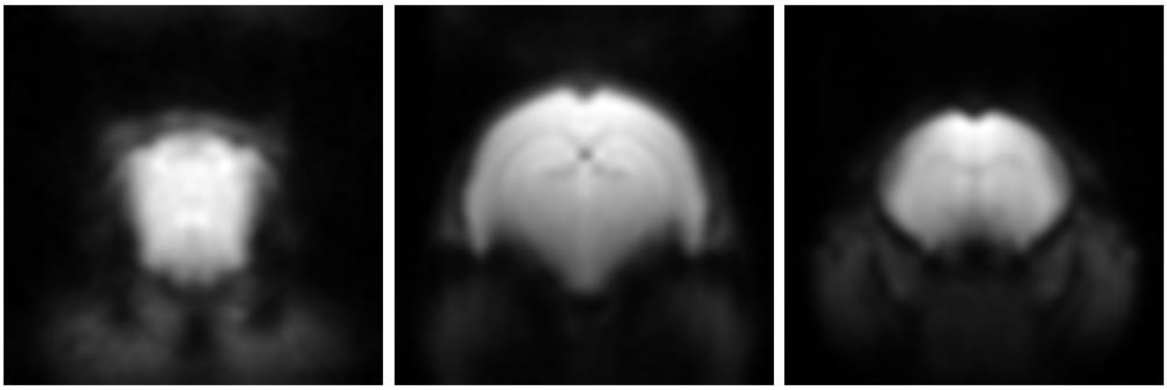

Supplementary Figure S1: Exemplary slices of the registered mean of all animals.
Bregma (from left to right): -6.48 mm, -2.06 mm, 1.42 mm.

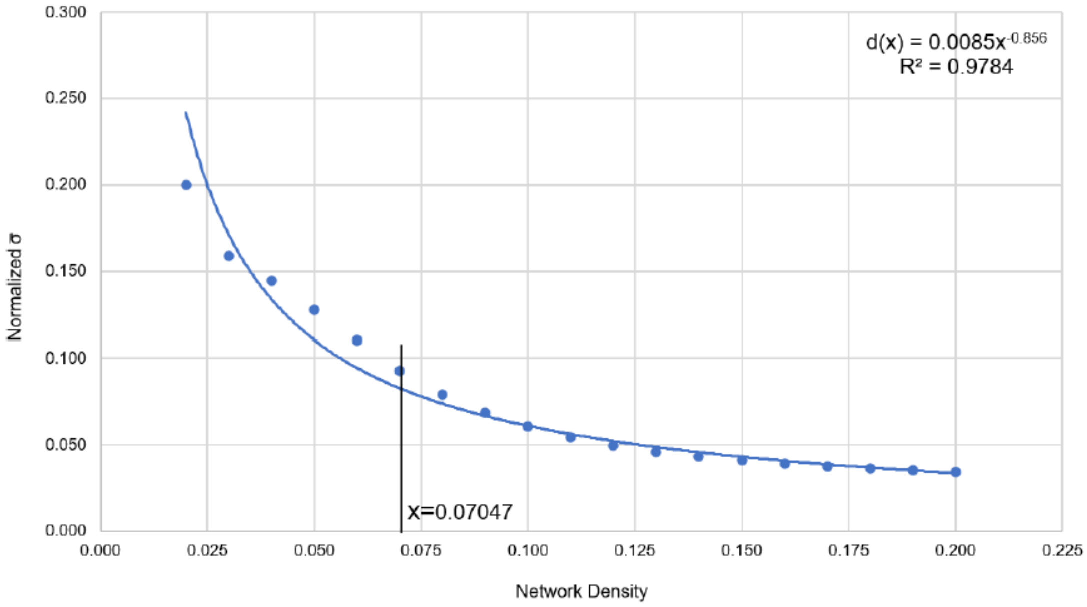

Supplementary Figure S2: Normalized small-world-index plotted as a function of the density.
Points represent data points; solid line represents fitted hyperbola. The maximum curvature (first derivative of hyperbola equated to -1) and resulting network density used for data analysis is 7%.

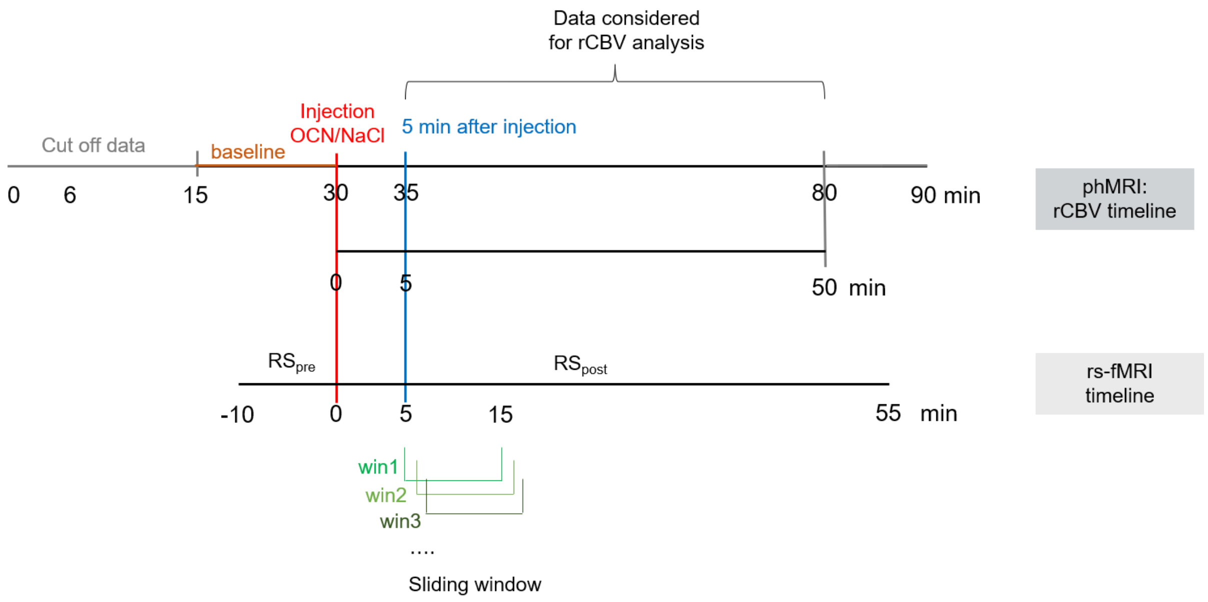

Supplementary Figure S3: Explanation of the rCBV and rs-fMRI timeline and period considered for data analysis.

The whole rCBV measurement had a duration of 90 min. Contrast agent was injected after 6 min recording time. After the equilibration time, i.e., 15 min after start of the recording (9 min after injection of contrast agent) the baseline started and lasted until 30 min (15 min in total). Data before the baseline were cut off and not considered for further analysis. Injection of OCN and NaCl, respectively, took place at 30 min after recording start. To achieve similar time description between RS and rCBV, the injection time point was set to 0 min. The last 10 min were cut off as well, as toward the end of the experiment, the real washout kinetics may differ from the exponentially fitted one which would influence the calculated rCBV signal. In the rs-fMRI timeline, the injection time point is also set to 0 min, with 10 min of RS_{pre} prior to injection, 5 min rest period after injection and the following RS_{post} measurement of in total 50 min. Sliding windows analysis was applied to ensure a dynamic analysis of the RS_{post} period, each window comprising 10 min, shifted by one minute along the measurement. The RS_{pre} measurement is one time window.

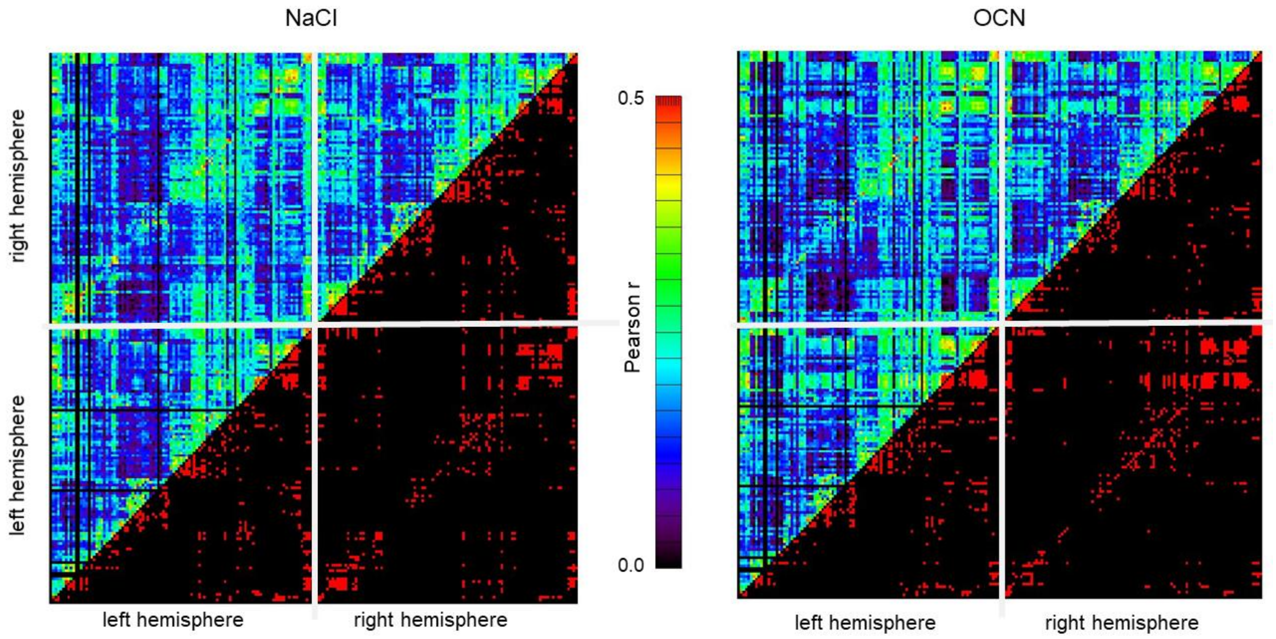

Supplementary Figure S4: Mean correlation matrices of NaCl group (left) and OCN group (right) before (upper left triangle) and after (lower right triangle) density thresholding of 7%.
Matrices are shown at the time point of interest, i.e. 34 min after substance injection.

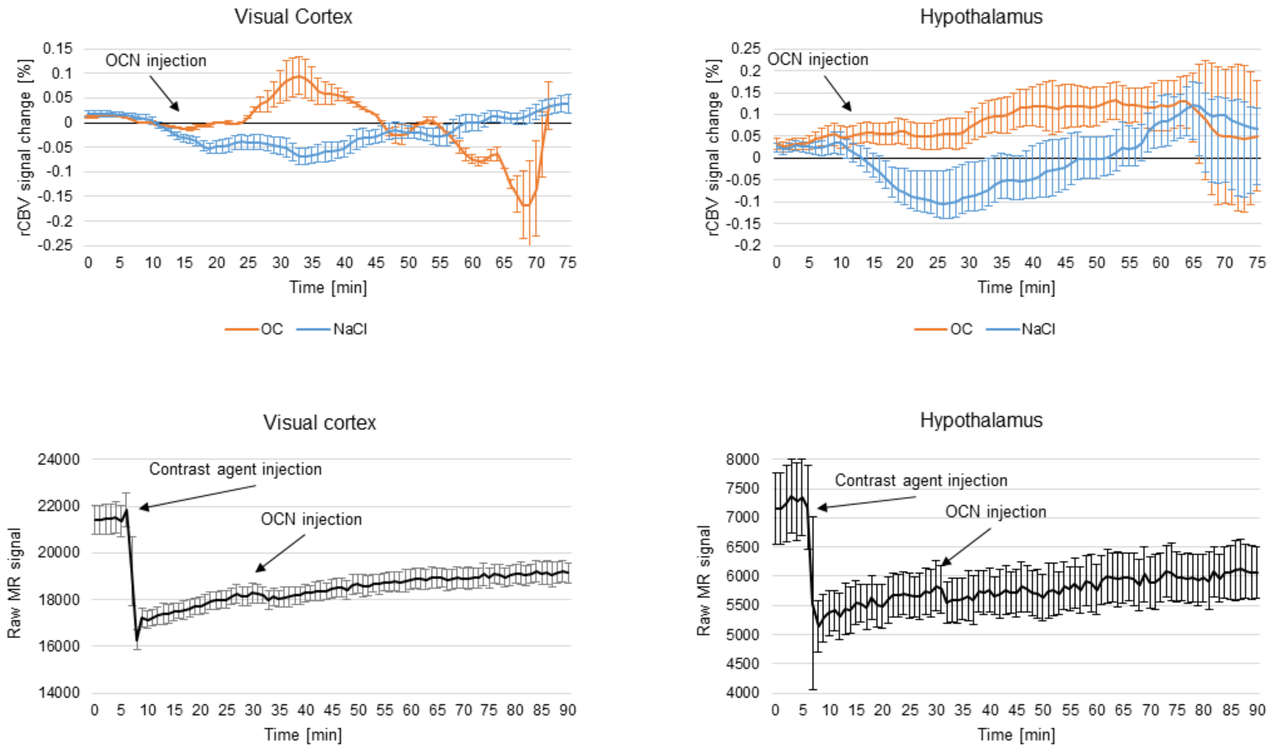

Supplementary Figure S5: Average rCBV time courses (group analysis; upper row) and average raw MR signal curves (OCN group, lower row) per brain region with standard deviation across region voxels.

Supplementary Table S1: Overview of the abbreviations of functional groups used in circular plots and their sub groups with their respective mean expression density of the genes Gpr37 and Gpr158. Values are displayed without left and right division.

Abbr.	Functional group	Brainstructure		Gpr37	Gpr158
OLF	Olfactory input	OLFlat	Nucleus of the lateral olfactory tract	0.057	1.160
OLF	Olfactory input	OLFar	Olfactory areas	1.235	2.754
OLF	Olfactory input	OLFbu	Olfactory bulb	1.014	1.479
BS	Brainstem	TRI	Spinal nucleus of the trigeminal nerve	1.877	1.024
BS	Brainstem	COCH	Cochlear nuclei	2.611	0.562
BS	Brainstem	SOL	Nucleus of the solitary tract	0.793	0.557
BS	Brainstem	MEDsr	Medulla sensory related	0.922	0.430
BS	Brainstem	MEDcran	Medulla cranial nerve nuclei	2.092	0.780
BS	Brainstem	MEDret	Medulla reticular formation	1.727	0.457
BS	Brainstem	MEDmr	Medulla motor related	2.144	0.489
BS	Brainstem	MEDrn	Medulla raphe nuclei	2.281	1.288
BS	Brainstem	PBN	Parabrachial nucleus	2.258	0.511
BS	Brainstem	PONsr	Pons sensory related	2.083	1.011
BS	Brainstem	PONmr	Pons motor related	2.086	0.712
BS	Brainstem	PONbr	Pons behavioral state related	1.703	1.539
BS	Brainstem	PONrn	Pons raphe nuclei	1.795	0.564
SI	Sensory input	TEG	Tegmental area	1.611	0.560
SI	Sensory input	COLinf	Inferior Colliculi	1.811	0.462
SI	Sensory input	COLsup	Superior Colliculi	0.797	0.720
SI	Sensory input	MBsr	Midbrain sensory related	1.117	0.380
SI	Sensory input	MBmr	Midbrain motor related	1.097	0.465
SI	Sensory input	PRT	Pretectal region	1.519	0.760
SI	Sensory input	MBbr	Midbrain behavioral state related	1.038	1.645
SI	Sensory input	MBrn	Midbrain raphe nuclei	0.987	0.344
TH_{sp}	Specific thalamus	THlgen	Lateral geniculate complex	1.533	1.175
TH_{sp}	Specific thalamus	THmgen	Medial geniculate complex	0.543	0.448
TH_{sp}	Specific thalamus	THvgen	Ventral geniculate complex	1.906	0.928
TH_{sp}	Specific thalamus	THlp	Lateral posterior nucleus of the thalamus	1.036	0.890
TH_{sp}	Specific thalamus	THpo	Posterior complex of the thalamus	0.997	1.511
TH_{sp}	Specific thalamus	THvm	Ventral medial nucleus of the thalamus	2.108	0.702
TH_{sp}	Specific thalamus	THvpol	Ventral posterolateral nucleus of the thalamus	2.651	0.191
TH_{sp}	Specific thalamus	THvpom	Ventral posteromedial nucleus of the thalamus	1.790	0.580
TH_{sp}	Specific thalamus	THldo	Lateral dorsal nucleus of thalamus	1.345	0.704
TH_{sp}	Specific thalamus	THmd	Mediodorsal nucleus of thalamus	0.553	1.880
TH_{sp}	Specific thalamus	THsub	Submedial nucleus of the thalamus	1.424	1.790

TH_{sp}	Specific thalamus	THva	Ventral anterior-lateral complex of the thalamus	1.924	0.528
TH_{sp}	Specific thalamus	THa	Anterior thalamus	0.477	0.294
TH_{unsp}	Unspecific thalamus	THlam	Intralaminar nuclei of the dorsal thalamus	1.123	1.741
TH_{unsp}	Unspecific thalamus	THpar	Parafascicular nucleus	1.437	1.176
TH_{unsp}	Unspecific thalamus	THmid	Midline group of the dorsal thalamus	0.266	0.514
TH_{unsp}	Unspecific thalamus	THper	Perireunensis nucleus	1.427	1.234
TH_{unsp}	Unspecific thalamus	THus	Thalamus unspecific	1.400	0.898
TH_{unsp}	Unspecific thalamus	THret	Reticular nucleus of the thalamus	2.607	0.117
CTXs	Sensory cortex	CTXs1bf	Primary somatosensory area barrel field	1.049	4.979
CTXs	Sensory cortex	CTXs1ll	Primary somatosensory area lower limb	0.909	2.625
CTXs	Sensory cortex	CTXs1mo	Primary somatosensory area mouth	1.722	6.149
CTXs	Sensory cortex	CTXs1no	Primary somatosensory area nose	1.936	5.788
CTXs	Sensory cortex	CTXs1tr	Primary somatosensory area trunk	0.900	2.877
CTXs	Sensory cortex	CTXs1ul	Primary somatosensory area upper limb	1.389	3.550
CTXs	Sensory cortex	CTXs1us	Primary somatosensory area unassigned	1.803	5.139
CTXs	Sensory cortex	CTXs2	Supplemental somatosensory area	0.991	5.106
CTXs	Sensory cortex	CTXvisc	Visceral area	0.662	6.994
CTXs	Sensory cortex	GUS	Gustatory areas	1.713	4.394
CTXs	Sensory cortex	CTXau	Auditory areas	0.932	1.435
CTXs	Sensory cortex	CTXvis	Visual areas	0.553	2.836
CTXm	Motor cortex	CTXm1	Primary motor area	1.570	3.515
CTXm	Motor cortex	CTXm2	Secondary motor area	1.117	4.236
CTXass	Association cortex	CTXtemp	Temporal association areas	0.985	3.048
CTXass	Association cortex	CTXpar	Posterior parietal association areas	0.652	2.667
CTXass	Association cortex	CTXains	Anterior Insula	1.610	4.156
CTXass	Association cortex	CTXpins	Posterior Insula	0.537	4.341
CTXass	Association cortex	CTXacg	Anterior cingulate area	1.239	3.297
CTXass	Association cortex	CTXrs	Retrosplenial area	1.156	3.066
CTXass	Association cortex	CTXil	Infralimbic area	1.752	2.190
CTXass	Association cortex	CTXpl	Prelimbic association cortex	0.900	3.265
CTXass	Association cortex	CTXfro	Frontal Pole	0.729	2.952
CTXass	Association cortex	CTXorb	Orbital area	0.803	5.199
CTXpl	Paralimbic cortex	CTXect	Ectorhinal area	1.145	1.880
CTXpl	Paralimbic cortex	CTXper	Perirhinal area	0.992	0.000
CTXpl	Paralimbic cortex	CTXent	Entorhinal area	0.724	1.677
CTXpl	Paralimbic cortex	PIR	Piriform area	0.882	3.430
LS	Limbic system	HAB	Habenulae	0.943	0.616
LS	Limbic system	SEP	Septum	1.380	0.630
LS	Limbic system	DB	Diagonal band nucleus	1.006	1.658

HPF	Hippocampal formation	HCad	Anteriodorsal hippocampus	1.956	1.873
HPF	Hippocampal formation	HCpd	Posteriodorsal hippocampus	1.282	1.833
HPF	Hippocampal formation	HCsd	Dorsal subiculum	0.925	0.863
HPF	Hippocampal formation	HCsv	Ventral subiculum	0.553	0.527
HPF	Hippocampal formation	HCv	Ventral hippocampus	0.661	0.869
HPF	Hippocampal formation	HCdg	Dentate gyrus	1.029	0.959
AMY	Amygdala	AMcor	Cortical amygdalar area	0.415	0.662
AMY	Amygdala	AMbal	Basolateral amygdalar nucleus	0.979	1.204
AMY	Amygdala	AMbam	Basomedial amygdalar nucleus	0.772	0.251
AMY	Amygdala	AMce	Central amygdalar nucleus	0.691	0.227
AMY	Amygdala	AMmed	Medial amygdalar nucleus	0.737	0.076
AMY	Amygdala	AMstr	Striatum-like amygdalar nuclei	0.424	0.646
AMY	Amygdala	AMbnst	Bed nuclei of the stria terminalis	0.886	0.179
HY	Hypothalamus	HYpvz	Periventricular hypothalamic zone	0.453	0.060
HY	Hypothalamus	HYpvr	Periventricular hypothalamic region	0.601	0.790
HY	Hypothalamus	HYlat	Lateral hypothalamus	1.154	0.255
HY	Hypothalamus	HYmed	Medial hypothalamus	0.769	0.464
HY	Hypothalamus	HYus	Hypothalamus unspecific	0.815	0.356
LO	Limbic output	ZI	Zona incerta	2.546	0.584
LO	Limbic output	PAG	Periaqueductal gray	0.851	0.414
LO	Limbic output	MAM	Mammillary body	1.018	0.022
BG	Basal ganglia	STRd	Dorsal Striatum	1.376	4.202
BG	Basal ganglia	STRv	Ventral Striatum	0.971	1.944
BG	Basal ganglia	CL	Claustrum	0.841	5.745
BG	Basal ganglia	PAL	Pallidum	1.793	0.821
CB	Cerebellum	CERhem	Cerebellar hemispheric region	1.395	0.653
CB	Cerebellum	CERver	Cerebellar vermis	1.368	0.512
CB	Cerebellum	CERflo	Cerebellar flocculus/paraflocculus	1.133	0.564
CB	Cerebellum	CERdn	Cerebellar deep nuclei	1.906	0.534
WM	White matter	CERfi	Cerebellum related fiber tracts	1.897	0.621

Supplementary Table S2: Overview of the assignment of brain structures to their functional groups with their respective values for the differences of the rCBV average maximum values of the OCN and NaCl group, hub score for NaCl, and hub score for OCN. For explanation of abbreviations see Supplementary Table S1. The suffix _r and _l, respectively, describes the right and left hemisphere.

Functional group	Brain region	rCBV average OCN_{peak}-$NaCl_{peak}$	Hub score NaCl	Hub score OCN
OLF_r	OLFlat_r	-0.021	5.704	8.931
OLF_r	OLFar_r	0.028	6.377	6.818
OLF_r	OLFbu_r	0.020	8.780	8.338
BS_r	TRI_r	-0.001	6.785	6.839
BS_r	COCH_r	-0.005	6.900	9.634
BS_r	SOL_r	0.038	5.858	3.043
BS_r	MEDsr_r	0.035	4.898	1.677
BS_r	MEDcran_r	0.007	4.140	1.692
BS_r	MEDret_r	0.022	4.978	2.174
BS_r	MEDmr_r	0.043	5.012	2.572
BS_r	MEDrn_r	0.012	3.623	1.439
BS_r	PBN_r	0.004	4.075	1.935
BS_r	PONsr_r	-0.010	5.081	1.885
BS_r	PONmr_r	-0.006	6.403	2.805
BS_r	PONbr_r	0.034	2.950	1.211
BS_r	PONrn_r	-0.016	4.260	2.465
SI_r	TEG_r	0.022	5.150	2.530
SI_r	COLinf_r	0.002	1.345	1.146
SI_r	COLsup_r	-0.006	3.039	0.623
SI_r	MBsr_r	0.002	2.992	0.389
SI_r	MBmr_r	-0.003	1.761	0.909
SI_r	PRT_r	0.005	0.693	0.377
SI_r	MBbr_r	0.010	0.673	1.234
SI_r	MBrn_r	0.007	0.890	0.142
TH_{sp}_r	THlgen_r	0.011	8.337	3.552
TH_{sp}_r	THmgen_r	0.001	5.964	4.308
TH_{sp}_r	THvgen_r	0.005	6.434	0.978
TH_{sp}_r	THlp_r	-0.005	1.699	1.226
TH_{sp}_r	THpo_r	0.015	1.736	0.910
TH_{sp}_r	THvm_r	0.015	1.612	1.805
TH_{sp}_r	THvpol_r	0.015	1.870	1.490
TH_{sp}_r	THvpom_r	0.023	1.370	1.207
TH_{sp}_r	THldo_r	0.019	0.356	1.976
TH_{sp}_r	THmd_r	0.024	1.950	3.041
TH_{sp}_r	THsub_r	-0.017	0.488	1.639
TH_{sp}_r	THva_r	0.022	0.834	1.940
TH_{sp}_r	THa_r	0.047	1.849	2.284
TH_{unsp}_r	THlam_r	0.017	1.731	1.494
TH_{unsp}_r	THpar_r	0.028	1.846	2.083

TH$_{unsp}$_r	THmid_r	-0.029	1.712	3.379
TH$_{unsp}$_r	THper_r	-0.020	1.690	4.211
TH$_{unsp}$_r	THus_r	0.011	0.421	1.155
TH$_{unsp}$_r	THret_r	0.008	2.060	2.649
CTXs_r	CTXs1bf_r	0.020	3.438	6.338
CTXs_r	CTXs1ll_r	0.015	0.894	1.589
CTXs_r	CTXs1mo_r	0.010	2.189	2.907
CTXs_r	CTXs1no_r	0.030	1.889	2.328
CTXs_r	CTXs1tr_r	0.014	3.520	2.588
CTXs_r	CTXs1ul_r	0.008	2.299	4.547
CTXs_r	CTXs1us_r	0.014	2.919	3.745
CTXs_r	CTXs2_r	-0.008	4.971	4.286
CTXs_r	CTXvisc_r	-0.008	2.060	2.914
CTXs_r	GUS_r	-0.010	2.586	3.451
CTXs_r	CTXau_r	0.027	3.621	3.153
CTXs_r	CTXvis_r	0.029	3.399	3.196
CTXm_r	CTXm1_r	0.009	3.195	5.028
CTXm_r	CTXm2_r	0.006	2.039	3.698
CTXass_r	CTXtemp_r	0.004	8.425	7.366
CTXass_r	CTXpar_r	0.032	11.668	7.777
CTXass_r	CTXains_r	0.001	5.017	4.077
CTXass_r	CTXpins_r	-0.029	5.796	6.449
CTXass_r	CTXacg_r	0.010	2.464	1.763
CTXass_r	CTXrs_r	0.011	5.935	5.310
CTXass_r	CTXil_r	0.017	4.298	5.795
CTXass_r	CTXpl_r	0.041	5.682	7.571
CTXass_r	CTXfro_r	0.039	3.388	3.041
CTXass_r	CTXorb_r	0.010	2.387	5.510
CTXpl_r	CTXect_r	-0.015	4.019	3.749
CTXpl_r	CTXper_r	-0.024	6.744	6.696
CTXpl_r	CTXent_r	0.000	6.674	7.310
CTXpl_r	PIR_r	-0.015	7.975	6.474
LS_r	HAB_r	-0.007	4.070	6.148
LS_r	SEP_r	-0.001	4.453	6.571
LS_r	DB_r	0.018	10.079	7.782
HPF_r	HCad_r	0.007	2.464	1.571
HPF_r	HCpd_r	0.009	1.775	2.179
HPF_r	HCsd_r	0.019	4.277	3.701
HPF_r	HCsv_r	-0.019	5.734	2.334
HPF_r	HCv_r	-0.015	5.696	4.035
HPF_r	HCdg_r	0.007	4.578	6.400
AMY_r	AMcor_r	0.015	10.798	10.557
AMY_r	AMbal_r	0.060	10.412	14.680
AMY_r	AMbam_r	-0.001	6.666	10.252
AMY_r	AMce_r	0.027	7.851	13.019
AMY_r	AMmed_r	0.022	6.183	6.485
AMY_r	AMstr_r	-0.002	3.226	3.681
AMY_r	AMbnst_r	0.018	2.116	0.821

HY_r	HYpvz_r	-0.013	0.531	0.641
HY_r	HYpvr_r	0.030	7.540	6.747
HY_r	HYlat_r	0.032	6.257	7.828
HY_r	HYmed_r	0.013	2.787	3.066
HY_r	HYus_r	0.014	4.328	3.643
LO_r	ZI_r	0.003	4.570	9.385
LO_r	PAG_r	-0.001	8.784	7.207
LO_r	MAM_r	0.034	6.795	9.820
BG_r	STRd_r	0.010	7.351	10.203
BG_r	STRv_r	0.016	7.498	9.982
BG_r	CL_r	-0.014	1.515	4.498
BG_r	PAL_r	0.014	1.404	4.342
CB_r	CERhem_r	-0.007	8.408	3.532
CB_r	CERver_r	0.019	7.340	4.789
CB_r	CERflo_r	-0.016	7.158	4.166
CB_r	CERdn_r	-0.023	6.691	3.665
OLF_l	OLFlat_l	0.000	0.000	0.000
OLF_l	OLFar_l	-0.008	6.578	5.892
OLF_l	OLFbu_l	-0.013	8.946	6.678
BS_l	TRI_l	0.037	7.044	7.408
BS_l	COCH_l	0.043	5.472	9.369
BS_l	SOL_l	0.011	5.706	3.390
BS_l	MEDsr_l	0.029	5.044	1.422
BS_l	MEDcran_l	0.019	6.285	4.979
BS_l	MEDret_l	0.000	5.428	2.136
BS_l	MEDmr_l	0.000	5.515	3.319
BS_l	MEDrn_l	0.040	0.000	0.000
BS_l	PBN_l	0.014	0.000	0.000
BS_l	PONsr_l	0.052	4.079	1.638
BS_l	PONmr_l	0.000	5.472	2.828
BS_l	PONbr_l	0.036	4.355	1.635
BS_l	PONrn_l	-0.004	0.000	0.000
SI_l	TEG_l	0.045	3.391	1.355
SI_l	COLinf_l	-0.002	1.846	1.835
SI_l	COLsup_l	0.002	3.359	0.701
SI_l	MBsr_l	0.024	4.002	0.642
SI_l	MBmr_l	-0.002	1.459	0.803
SI_l	PRT_l	-0.006	0.378	1.039
SI_l	MBbr_l	0.014	0.754	2.316
SI_l	MBrn_l	-0.005	0.564	0.192
TH$_{sp}$_l	THlgen_l	-0.006	8.942	3.284
TH$_{sp}$_l	THmgen_l	0.020	6.863	2.786
TH$_{sp}$_l	THvgen_l	0.011	7.379	1.182
TH$_{sp}$_l	THlp_l	0.011	1.305	1.952
TH$_{sp}$_l	THpo_l	0.019	1.749	1.754
TH$_{sp}$_l	THvm_l	-0.002	1.597	3.219
TH$_{sp}$_l	THvpol_l	0.016	1.423	1.941
TH$_{sp}$_l	THvpom_l	0.022	1.246	1.136

TH$_{sp}$_l	THldo_l	-0.003	0.244	1.722
TH$_{sp}$_l	THmd_l	0.031	0.692	3.420
TH$_{sp}$_l	THsub_l	-0.007	0.661	2.251
TH$_{sp}$_l	THva_l	0.010	0.587	2.757
TH$_{sp}$_l	THa_l	0.012	1.542	2.593
TH$_{unsp}$_l	THlam_l	-0.001	1.046	2.159
TH$_{unsp}$_l	THpar_l	0.025	1.447	1.972
TH$_{unsp}$_l	THmid_l	0.000	1.599	1.461
TH$_{unsp}$_l	THper_l	0.025	1.459	4.012
TH$_{unsp}$_l	THus_l	0.009	0.229	1.534
TH$_{unsp}$_l	THret_l	-0.002	0.000	0.000
CTXs_l	CTXs1bf_l	0.011	3.634	6.556
CTXs_l	CTXs1ll_l	0.019	0.549	2.528
CTXs_l	CTXs1mo_l	0.004	2.110	2.982
CTXs_l	CTXs1no_l	-0.005	1.844	1.882
CTXs_l	CTXs1tr_l	0.005	4.474	1.707
CTXs_l	CTXs1ul_l	0.007	2.323	2.495
CTXs_l	CTXs1us_l	-0.027	2.931	2.844
CTXs_l	CTXs2_l	0.009	6.336	1.515
CTXs_l	CTXvisc_l	0.024	4.129	2.151
CTXs_l	GUS_l	0.018	2.906	3.409
CTXs_l	CTXau_l	0.000	2.523	2.278
CTXs_l	CTXvis_l	0.022	2.049	2.534
CTXm_l	CTXm1_l	0.003	4.199	7.413
CTXm_l	CTXm2_l	0.025	3.933	2.379
CTXass_l	CTXtemp_l	0.006	5.272	7.794
CTXass_l	CTXpar_l	0.034	9.269	7.240
CTXass_l	CTXains_l	0.023	5.296	3.499
CTXass_l	CTXpins_l	0.036	8.940	5.632
CTXass_l	CTXacg_l	0.014	2.324	1.455
CTXass_l	CTXrs_l	0.008	4.525	3.428
CTXass_l	CTXil_l	-0.003	4.111	5.008
CTXass_l	CTXpl_l	0.055	3.481	7.839
CTXass_l	CTXfro_l	0.016	6.184	3.505
CTXass_l	CTXorb_l	0.032	6.774	9.630
CTXpl_l	CTXect_l	0.014	4.398	4.185
CTXpl_l	CTXper_l	0.000	6.890	5.323
CTXpl_l	CTXent_l	0.006	5.821	6.258
CTXpl_l	PIR_l	0.016	8.059	7.285
LS_l	HAB_l	0.008	2.856	8.737
LS_l	SEP_l	-0.009	0.000	0.000
LS_l	DB_l	0.020	5.854	9.859
HPF_l	HCad_l	-0.004	2.890	2.920
HPF_l	HCpd_l	0.012	2.173	2.394
HPF_l	HCsd_l	0.006	3.703	5.255
HPF_l	HCsv_l	0.003	6.298	1.715
HPF_l	HCv_l	-0.005	3.134	2.950
HPF_l	HCdg_l	-0.004	3.070	9.169

AMY_l	AMcor_l	-0.006	7.673	11.784
AMY_l	AMbal_l	-0.021	9.145	12.770
AMY_l	AMbam_l	-0.035	6.859	11.083
AMY_l	AMce_l	0.013	8.225	11.572
AMY_l	AMmed_l	-0.016	7.288	9.872
AMY_l	AMstr_l	-0.008	2.705	2.937
AMY_l	AMbnst_l	-0.017	2.968	0.894
HY_l	HYpvz_l	-0.010	0.701	0.840
HY_l	HYpvr_l	0.030	6.745	5.106
HY_l	HYlat_l	0.070	5.788	7.399
HY_l	HYmed_l	0.010	3.249	2.605
HY_l	HYus_l	0.009	4.309	3.723
LO_l	ZI_l	0.028	5.161	10.531
LO_l	PAG_l	0.002	8.243	8.359
LO_l	MAM_l	0.029	8.779	10.040
BG_l	STRd_l	0.005	7.525	10.526
BG_l	STRv_l	0.009	7.537	10.993
BG_l	CL_l	0.034	1.237	5.924
BG_l	PAL_l	-0.006	1.195	4.014
CB_l	CERhem_l	0.013	5.849	4.161
CB_l	CERver_l	0.009	7.946	4.821
CB_l	CERflo_l	0.008	7.148	4.346
CB_l	CERdn_l	-0.039	7.224	3.874
